# Women presenting to emergency departments with vaginitis should be offered single‐dose empirical treatment

**DOI:** 10.1111/acem.70026

**Published:** 2025-03-26

**Authors:** David A. Talan, Omai B. Gardner, Brett A. Faine

**Affiliations:** ^1^ Department of Emergency Medicine, UCLA Health The David Geffen School of Medicine at the University of California at Los Angeles Los Angeles California USA; ^2^ Department of Pathology and Laboratory Medicine, UCLA Health The David Geffen School of Medicine at the University of California at Los Angeles Los Angeles California USA; ^3^ Department of Pharmacy Practice and Science University of Iowa College of Pharmacy Iowa City Iowa USA

Bacterial vaginosis (BV), *Candida*, and *Trichomonas vaginalis* (TV) are the most common causes of vaginitis. These infections are associated with various untoward consequences including increased risk of acquisition of the others, transmission of HIV, *Neisseria gonorrhoeae*, *Chlamydia trachomatis*, and herpes simplex virus‐2 as well as adverse reproductive and mental health outcomes, and cervical cancer risk.^1^ Vaginitis disproportionately affects ethnic minorities and those with lower formal educational attainment and/or experiencing poverty, the very groups most likely to seek episodic care in emergency departments (EDs).^2^, ^3^, ^4^ Current U.S. Centers for Disease Control and Prevention (CDC) sexually transmitted infections (STIs) treatment guidelines for vaginitis do not provide an effective approach for many of these women, resulting in missed and delayed diagnoses and treatment.^1^ We propose an empirical, single‐dose, point‐of‐care treatment strategy, novel to the U.S. but evidence‐based and consistent with international guidelines, to address existing deficiencies in availability of inexpensive and accurate point‐of‐care diagnosis, and substantial barriers to medication adherence and follow‐up commonly faced by women from disadvantaged groups who frequently seek help in EDs.^5^, ^6^, ^7^

Vaginitis is clinically diagnosed by complaints of itching, burning, and odor accompanied by vaginal discharge. However, providing effective treatment is challenging because of limitations to accurately diagnose the cause of vaginitis at the time of a woman's visit. Conventional microscopy (Gram stain and wet mount), which is typically available in EDs, in addition to being time‐consuming, is strikingly insensitive for the diagnosis of BV, TV, and *Candida*, particularly with coinfections.^8^, ^9^, ^10^, ^11^, ^12^ For example, one study of 1740 women with vaginitis (BV, 783; *Candida*, 523; TV, 122; and mixed BV/*Candida*, 185) comparing standard microscopy to nucleic acid amplification testing (NAAT; using reference standards of culture for TV and *Candida* and Nugent criteria for BV) reported sensitivities for microscopy to detect BV, TV, and *Candida* were 76.8%, 68.6%, and 56.9%, respectively.^8^ For NAAT, sensitivities were 92.8%, 96.5%, and 90.2%, respectively. Specificities for both microscopy and NAAT were generally >90%. For microscopy detection of coinfections, sensitivities dropped to 10%–20%. Considering single and multiple infections, using microscopy, as many as one‐quarter to one‐half of infections are missed overall. CDC STI guidelines currently acknowledge microscopy's very poor sensitivity, recommending NAAT TV and *Candida* culture testing (or empirical treatment for *Candida*) if microscopy is negative.^1^

Currently, the only point‐of‐care NAAT for BV, *Candida*, and TV, the Cepheid Xpert Express MVP PCR test, with a minimum laboratory assay turnaround time of 60 min but longer in practice, is not generally available in U.S. EDs (based on a survey of 12 U.S. EDs in our *EMERGE*ncy ID NET research network [https://www.emergencyidnet.org/] and communication with Cepheid Company). Urgent care facilities likely have even less laboratory support. Use of microscopy testing leads to missed diagnoses and inadequate or delayed treatment, with persistence of symptoms additionally contributing to these women's disabilities as well as risks of adverse health outcomes. In many centers, NAATs can be ordered as send‐out tests; however, they are expensive, covered by most but not all insurance, and require follow‐up, which pose real obstacles for many women who are uninsured or underinsured, are unhoused, and lack a telephone and transportation. Further, follow‐up of send‐out test results, counseling, and prescriptions take time, which has a potential operational impact on ED workflow. The consequences of inadequate treatment and inefficient testing approaches could be mitigated in these circumstances by an affordable, single‐dose, empirical point‐of‐care treatment strategy.

The 2021 U.S. CDC STI guidelines primarily recommend a 7‐day 500‐mg twice‐daily oral metronidazole regimen to treat BV and TV in women and 150‐mg single‐dose oral fluconazole to treat candidal infection (Table 1).^1^ For treatment of women with TV, a 7‐day 500 mg twice daily metronidazole regimen has been found to be associated with higher proportion of patients achieving 4‐week cure than the 2‐g single dose.^13^ Adherence is difficult due to this regimen's long duration and metronidazole's unpleasant metallic taste and frequent gastrointestinal side effects.^14^ As opposed to sexual health clinics, regulatory barriers obviate point‐of‐care ED medication dispensing, thus requiring the patient to go to a pharmacy to fill their prescription. One study found that patient adherence to multidose oral metronidazole treatment of BV was 50%–68%.^15^ Women of ethnic minorities and low socioeconomic status are at greatest risk of medication nonadherence.^16^

**TABLE 1:** U.S. CDC, European International Union/WHO, and U.K. guidelines for treatment of BV and trichomoniasis.

	BV		Trichomoniasis	
	Recommended	Alternative	Recommended	Alternative
U.S. CDC^1^	Metronidazole 500 mg orally BID × 7 days; *or* Metronidazole gel 0.75% 5 g intravaginally daily × 5 days; *or* Clindamycin cream 2% 5 g intravaginally daily × 7 days	Tinidazole 2 g orally single dose QD × 2 days; *or* Tinidazole 1 g orally QD × 5 days *or* Secnidazole 2 g orally single dose; *or* Clindamycin 300 mg orally BID × 7 days; *or* Clindamycin 100 mg intravaginally QD × 3 days	Metronidazole 500 mg orally BID × 7 days	Tinidazole 2 g orally single dose
European International Union/WHO^5^	Metronidazole 400–500 mg orally BID × 5–7 days; *or* Metronidazole 2 g orally single dose *or* Metronidazole gel 0.75% 5 g intravaginally QD × 5 days; *or* Clindamycin cream 2% 5 g intravaginally QD × 7 days	Tinidazole 2 g orally single dose *or* Tinidazole 1 g orally QD × 5 days *or* Metronidazole 2 g orally single dose *or* Clindamycin 300 mg orally BID × 7 days	Tinidazole 2 g orally single dose *or* Metronidazole 400–500 mg orally BID × 5–7 days; *or* Metronidazole 2 g orally single dose	
United Kingdom^6^, ^7^	Metronidazole 400 mg orally BID × 5–7 days; *or* Metronidazole 2 g orally in a single dose *or* Metronidazole gel 0.75% 5 g intravaginally daily × 5 days; *or* Clindamycin cream 2% 5 g intravaginally daily × 7 days	Tinidazole 2 g orally single dose *or* Clindamycin 300 mg orally BID × 7 days	Metronidazole 400–500 mg orally BID × 5–7 days	Metronidazole 2 g orally single dose

Abbreviations: BID, two times a day; CDC, Centers for Disease Control and Prevention; QD, once a day; WHO, World Health Organization.

In light of these practical realities, empirical single‐dose oral tinidazole treatment of both BV and TV (with single‐dose fluconazole) would offer a patient‐centered solution to optimize the chance for first‐time cure, particularly for women challenged by medication adherence and follow‐up capability. Currently, the 2021 U.S. CDC STI guidelines recommend 2‐g single‐dose tinidazole (as four 500‐mg tablets) for TV treatment and 2 g on Days 1 and 2 as an alternative BV treatment option.^1^ Due to ED dispensing limitations, the next day's dose would require the patient to fill a prescription. However, 2‐g single‐dose tinidazole is already recommended as an alternative treatment of both BV and TV by the European International Union against Sexually Transmitted Infections (IUSTI) and World Health Organization (WHO) and the British Association for Sexual Health and HIV (BASHH) U.K. guidelines (Table 1).^5^, ^6^, ^7^ Tinidazole's plasma half‐life is 12–14 h, which is longer than metronidazole's (~8 h), allowing for a shorter treatment course, and its safety profile is less adverse, with metallic taste, nausea, anorexia, and constipation associated with longer duration as opposed to single‐dose treatment.^17^ TV also exhibits less resistance to tinidazole than metronidazole, demonstrating both lower in vitro minimum lethal concentrations and a high clinical efficacy rate for patients with metronidazole‐refractory TV infection.^18^

In 2017, the U.S. Food and Drug Administration (FDA) approved 2‐g single‐dose oral secnidazole (two 1‐g packets) for treatment of BV and TV, and this is currently listed as an alternative BV treatment option in U.S. CDC guidelines.^1^, ^19^ Table 2 shows the results of clinical trials among women with symptomatic vaginitis comparing metronidazole, tinidazole, and secnidazole, including single‐dose regimens, to treat BV and TV.^20^, ^21^, ^22^, ^23^, ^24^, ^25^, ^26^ For BV, when compared with metronidazole 500 mg BID for 7 days, both 2‐g single‐dose tinidazole and 2‐g single‐dose secnidazole demonstrated similar cure rates to metronidazole.^22^, ^25^ Another trial comparing single doses of secnidazole, tinidazole, and metronidazole to treat BV found that tinidazole had a significantly greater clinical cure rate than metronidazole but secnidazole did not, further supporting the efficacy of single‐dose tinidazole.^26^ Across all trials, for BV, 2‐g single‐dose tinidazole demonstrated clinical cure rates of 83%–100%.^20^, ^21^, ^22^, ^23^, ^24^, ^25^, ^26^

**TABLE 2:** Comparative clinical trials of tinidazole, secnidazole, and metronidazole treatment for symptomatic women diagnosed with BV and trichomoniasis.

Study (year)	Study design	Vaginitis etiology	Treatment regimens (number of assigned participants per group)	Outcomes	Cure rates	*p*‐value
Anjaneyulu et al. (1977)^20^	Open‐label, RCT	Trichomoniasis	MTZ 2 g one dose (50) TZD 2 g one dose (50)	12‐day clinical cure	MTZ—50% TDZ—84%	<0.01
				12‐day microbiological cure (wet mount)	MTZ—64% TDZ—94%	<0.01
Mohanty and Deighton (1987)^21^	Open‐label RCT	BV	MTZ 2 g one dose (82) NZ 2 g one dose (100) TZD 2 g one dose (98)	7‐day clinical cure	MTZ—89% NZ—90% TDZ—83%	NS
				7‐day microbiological cure (culture)	MTZ −79% NZ—88% TDZ—92%	NS
Buranawarodomkul et al. (1990)^22^	Open‐label RCT	BV	MTZ 500 mg BID 7 days (50) TDZ 2 g one dose (50)	1–2 week clinical cure^a^	MTZ—92% TDZ—86%	NS
Schindler et al. (1991)^23^	Open‐label RCT	BV	MTZ vaginal gel BID 5 days (84) TDZ 2 g one dose (75)	10–14 day clinical cure	MTZ—82% TDZ—84%	NS
O‐Prasertsawat and Jetsawangsri (1992)^24^	Double‐blind, double‐ dummy, RCT	Trichomoniasis	MTZ 1.6 g single‐day split dose (67) TZD 2 g one dose (65)	16‐day microbiological cure (culture)	MTZ—98.5% TDZ—100%	NS
Bohbot et al. (2010)^25^	Double‐blind, double‐dummy, RCT	BV	MTZ 500 mg BID 7 days (287) SEC 2 g 1 dose (290)	28‐day clinical and microbiological cure (Amsel criteria and Nugent score)	MTZ—59.5% SEC—60.1%	NS
Thulkar et al. (2012)^26^	Open‐label, RCT	BV	MTZ 2 g one dose (86) TZD 2 g one dose (86) SEC 2 g one dose (86) ORN 1.5 g one dose (86)	7‐day clinical cure (Amsel criteria)	MTZ—88.4% TDZ—100% SEC—90.7% ORN—100%	TDZ and ORN vs. MTZ, *p* < 0.01 SEC vs. MTZ, NS

Abbreviations: BID, two times a day; MTZ, metronidazole; NZ, nimorazole; ORN, ornidazole; RCT, randomized controlled trial; SEC, secnidazole; TDZ, tinidazole.

^a^ Microbiological cure by rates of individual posttreatment Amsel criteria were reported and all were found to be statistically similar between treatment groups.

Unlike FDA guidance for comparative noninferiority trials to evaluate new antibiotics to treat other common infections, guidance for BV antimicrobial approval specifies a placebo‐controlled superiority trial.^27^ Tinidazole was FDA‐approved in 2004 based on a placebo‐controlled trial that demonstrated efficacy of both 2 g daily for 2 days and for 5 days.^28^ Earlier placebo‐controlled trials and many comparative studies of a single 2‐g dose of tinidazole were conducted outside the United States and had a different design than specified in U.S. FDA guidance to evaluate BV treatments, which may explain the divergence of U.S. and international treatment recommendations (i.e., 2 g daily for 2 days vs. single dose).^29^ Tinidazole has gone off‐patent, so no commercial interest now exists for FDA approval of single‐dose treatment. However, as shown in Table 2, single‐dose tinidazole has compared favorably to both 7‐day twice‐daily metronidazole and single‐dose secnidazole, regimens that have met FDA guidance criteria, and tinidazole has the endorsement of agencies outside the United States, supporting its off‐label use.^5^, ^6^, ^7^

Secnidazole is substantially more expensive than metronidazole and tinidazole, well beyond the means of many women. Based on GoodRx.com (accessed March 1, 2025) for major chain pharmacies at multiple U.S. cities, the current cost of metronidazole 500 mg twice daily for 7 days and of 2‐g single‐dose tinidazole is about $10; the cost of 2‐g single‐dose secnidazole is about $300. Tinidazole is widely available in U.S. pharmacies and could be easily stocked in EDs. Author review of Medicaid Preferred Drug Lists (PDL; conducted during December 2024) found that tinidazole is included in 25 states presently (nine additional states require prior authorization); metronidazole is included in 44 states and secnidazole in two states (five states have no publicly accessible online PDL and 11 do not specifically address these treatments). Medicaid would cover costs of ED testing and treatment like many other insurances, through bundled payments, which come against the hospital's bottom line.

Regardless of the diagnostic and treatment approach to vaginitis, it should be stressed that a careful history for STI risk and pelvic examination remain important since vaginal discharge and discomfort can also be symptoms of gonococcal and chlamydial cervicitis and pelvic inflammatory disease, and broader STI screening may be indicated. As opposed to NAAT for vaginitis pathogens, gonococcal and chlamydial NAAT is relatively inexpensive and consistently covered by insurance, and laboratories are also required to report these infections to local public health agencies, which helps ensure proper follow‐up care. Women with STI risk suspected of TV should be advised of the need for abstinence pending partner evaluation and treatment, which could include expedited partner therapy (EPT) with 2‐g single‐dose metronidazole or tinidazole in states that allow it. Although not recommenderd in the last U.S. CDC STI guidelines, a recent placebo‐controlled clinical trial demonstrated that concurrent treatment of the male partner of a woman with BV significantly reduced the incidence of recurrent vaginitis within 12 weeks.^1^, ^30^ Of note, metronidazole is no longer contraindicated in pregnancy, whereas tinadazole and fluconazole are contraindicated in the first 3 months of pregnancy and, therefore, would require pregnancy screening. Secnidazole has not been assigned a pregnany rating.

Women who seek care and can follow up at sexual health clinics and primary care offices are different than many from disadvantaged groups for whom there may be only one opportunity to address their symptoms when seen in the ED. Empirical treatment of vaginitis carries risks of overtreatment, although use of a single‐dose regimen would minimize stewardship concerns for collateral adverse antibiotic effects. Further, overall treatment effectiveness must be considered in the context of a woman's specific circumstances and needs, such as CDC‐endorsed empirical EPT treatment for gonorrhea and chlamydia, and in the case of sexual assault, for which compliance with follow‐up visits is acknowledged to be poor.^1^

For women with the clinical diagnosis of vaginitis, particularly in EDs where point‐of‐care NAAT testing is not available, we believe empirical treatment with 2‐g single‐dose tinidazole for BV and TV and 150‐mg single‐dose fluconazole for *Candida* should be offered through shared decision making. Reliance on microscopy testing poses a substantial risk of underdiagnosis and undertreatment with subsequent persistent symptoms, additional health care visits, and risk of transmission. Alternatively, if the patient believes she can comply, the CDC‐recommended second‐day 2‐g tinidazole dose or 7‐day 500‐mg twice‐daily metronidazole regimen can also be prescribed empirically to treat BV and TV, and women with a history of recurrent or persistent *Candida* infection can be prescribed a Day 4 and 7 fluconazole dose.^1^ Whether empirical or microscopy/point‐of‐care NAAT‐directed treatment is provided, follow‐up should be recommended for patients whose symptoms persist beyond 1 week, at which time further diagnostic testing can be considered.

## CONFLICT OF INTEREST STATEMENT

The authors declare no conflicts of interest.

## Data Availability

Data sharing not applicable to this article as no datasets were generated or analysed during the current study.
